# Are diagnostic criteria for acute malnutrition affected by hydration status in hospitalized children? A repeated measures study

**DOI:** 10.1186/1475-2891-10-92

**Published:** 2011-09-13

**Authors:** Martha K Mwangome, Gregory Fegan, Andrew M Prentice, James A Berkley

**Affiliations:** 1 KEMRI/Wellcome Trust Research Programme, Centre for Geographic Medical Research Coast, PO Box 230, Kilifi, 80108, Kenya; 2 MRC International Nutrition Group. London School of Hygiene and Tropical Medicine, London, UK and MRC Keneba, The Gambia; 3 Centre for Clinical Vaccinology & Tropical Medicine, University of Oxford, Oxford, UK; 4 Medical Research Council Laboratories, Keneba Field Station, The Gambia

**Keywords:** Malnutrition, dehydration, MUAC, weight-for-length, anthropometry, children, Africa

## Abstract

**Introduction:**

Dehydration and malnutrition commonly occur together among ill children in developing countries. Dehydration (change in total body water) is known to alter weight. Although muscle tissue has high water content, it is not known whether mid-upper arm circumference (MUAC) may be altered by changes in tissue hydration. We aimed to determine whether rehydration alters MUAC, MUAC Z score (MUACz), weight-for-length Z-score (WFLz) and classification of nutritional status among hospitalised Kenyan children admitted with signs of dehydration.

**Study procedure:**

We enrolled children aged from 3 months to 5 years admitted to a rural Kenyan district hospital with clinical signs compatible with dehydration, and without kwashiorkor. Anthropometric measurements were taken at admission and repeated after 48 hours of treatment, which included rehydration by WHO protocols. Changes in weight observed during this period were considered to be due to changes in hydration status.

**Results:**

Among 325 children (median age 11 months) the median weight gain (rehydration) after 48 hours was 0.21 kg, (an increase of 2.9% of admission body weight). Each 1% change in weight was associated with a 0.40 mm (95% CI: 0.30 to 0.44 mm, p < 0.001) change in MUAC, 0.035z (95% CI: 0.027 to 0.043z, P < 0.001) change in MUACz score and 0.115z (95% CI: 0.114 to 0.116 z, p < 0.001) change in WFLz. Among children aged 6 months or more with signs of dehydration at admission who were classified as having severe acute malnutrition (SAM) at admission by WFLz <-3 or MUAC <115 mm, 21% and 19% of children respectively were above these cut offs after 48 hours.

**Conclusion:**

MUAC is less affected by dehydration than WFLz and is therefore more suitable for nutritional assessment of ill children. However, both WFLz and MUAC misclassify SAM among dehydrated children. Nutritional status should be re-evaluated following rehydration, and management adjusted accordingly.

## Introduction

Malnutrition is a leading cause of death among children under 5 years in sub-Saharan Africa [1]. Two forms of severe acute malnutrition (SAM) are recognized: severe wasting and kwashiorkor. The principle diagnostic criteria for SAM are based on weight for length Z score (WFLz) [2] and mid-upper arm circumference (MUAC) [3-6].

Dehydration due to gastroenteritis and other illnesses is common amongst children admitted to hospital in sub-Saharan Africa [7]. Diarrhoea and acute malnutrition often occur simultaneously [8]. In most clinical guidelines, dehydration is assessed by percent change in weight. Severe dehydration is commonly regarded as a weight change of more than 10% [9]. Hydration status and the contents of the gastrointestinal tract may cause short term variation in weight, due to changes in total body water, thus WFLz can be influenced by hydration status in sick children.

MUAC measures the circumference of the upper arm which is made up of lean muscle, fat tissue, [4] as well as a cross section of bone and blood vessels. MUAC is therefore a proxy measure of protein and lipid reserves. Lean muscle tissue contains about 75% water and therefore MUAC could conceivably be affected by tissue dehydration. However, this has not been established.

Among clinical signs of dehydration, three (prolonged capillary refill time, abnormal skin turgor and abnormal respiratory pattern) are regarded as the most reliable in diagnosing dehydration [10]. Current WHO guidelines, classify children as having severe, 'some' or 'no' dehydration, using signs including unconsciousness/lethargy/restlessness, sunken eyes, inability to drink, thirst, abnormal skin turgor [9]. Some of these signs, such as sunken eyes and abnormal skin turgor may also occur as features of acute wasting.

We aimed to determine whether rehydration alters MUAC and WFLz among children admitted to hospital with signs compatible with dehydration, and evaluate the potential for misclassification of nutritional status.

## Methods

### Study location

The study was conducted from February 2009 through August 2009 at Kilifi District Hospital, located in a rural area at the Kenyan Coast, approximately 65 kilometres north of Mombasa. Kilifi is the second poorest district in Kenya with 67% of the population living in poverty [11]. Under nutrition is endemic with 25% of the children under five underweight (WFAz <-2)[12] and hospital records indicate that approximately 500 cases of severe acute malnutrition admitted at the hospital every year, about 20% of whom are HIV exposed or infected.

### Study design

Using a before-after study design, anthropometric measurements were taken first at admission (before the hydration therapy) and again at 48 hours after admission.

### Study participants

We recruited children admitted to the hospital aged between 3 months and 5 years with one or more of the following clinical signs of dehydration at admission: reduced skin turgor, sunken eyes, delayed capillary refill (>3 seconds), weak pulse, deep breathing, or a palpable limb temperature gradient [9]. At admission, the admitting clinician assessed the clinical signs and described the initial diagnosis of the children. Age was ascertained through individual health cards or previous hospital records. Clinical details of all patients admitted were immediately recorded onto a database. Eligible patients were automatically flagged by this system, based on the inclusion criteria. These children were identified and their MUAC, weight and height were measured and recorded. Measures were repeated after 48 hours. Rehydration and other care were provided by experienced research clinicians not directly involved in this study. Management, following the current WHO guidelines, included special fluids, feeds and procedures for SAM [9]. Children were excluded from the study if they had kwashiorkor (bipedal oedema with or without dermatosis, a protruding belly or hair changes) since short-term changes in weight may be due to changes in oedema, or if they died or were discharged before the second set of measurements.

### Study Procedures

We trained two observers to take anthropometric measures according to procedures given in the United Nations guide: "How to Weigh and Measure Children" [13]. Weight was measured to the nearest 10 g using a Tanita 1582 digital weighing scale, which was quality controlled every morning using standard weights and the readings plotted on a calibration chart. For children below 24 months, length was taken using a wooden length board to the nearest 1 mm using a standard UNICEF design [14]. Height, which was taken among children 24 months or more, was measured using a wooden stadiometer to the nearest 1 mm using a mounted tape measure according to the UNICEF design. MUAC was measured on the left arm of the child using a tape to the nearest 2 mm using a dedicated insertion tape (Teaching Aid and Low Cost (TALC) St Albans, UK).

We established the inter-observer variation between the two observers. Each observer took MUAC, weight and height/length measures among children aged between 3 months and 5 years admitted to the paediatric ward. The two sets of measures were taken within 30 minutes and were blinded from the other observer.

### Ethical consideration

The study was approved by the Kenyan National Scientific and Ethical Review Committees (SCC 1418). Verbal consent was given by the caregiver of the child during the second measurement.

### Statistical methods

For the inter-observer study, the required sample size was determined using the sample size design for reliability studies [15,16]. This method calculates sample sizes for studies where the intra class correlation (ICC) is used as a measure of reliability.

For the repeated measures study, we calculated the sample size for paired studies using the formula described by Fleiss L. J et al [17], which is dependent on the difference between means and the within-group variability of individual measurements. To establish mean and standard deviation values for this population, we used historical MUAC data of hospitalised children from birth to 5 years collected between the years 2000 to 2005. We assumed that an arbitrary difference of 0.5 cm for MUAC would be clinically relevant.

Calculated sample sizes were n = 53 for the inter-observer study, and n = 285 for the repeated measures study each giving a 90% power of rejecting the null hypothesis of no difference at the 0.05 level of significance. In order to allow for drop out of children from the study, we rounded up these numbers to n = 60 and n = 300 children for the inter-observer and repeated measures study respectively.

Anthropometric Z score calculations for weight-for-length (WFLz), weight-for-age (WFAz), height-for-age (HFAz) and MUAC-for-age (MUACz) were computed using the 2006 WHO standards [2]. Statistical analysis was carried out using STATA version 11.0 (STATA Corp, College Station, Texas).

Rehydration solutions (ORS and ReSoMal), and the starter milk formula (F-75) used for stabilization of severely malnourished children provide hardly any energy and little protein, making deposition of lean or fat tissue negligible and unlikely to influence measures. Thus, we assumed that changes in weight and MUAC among acutely ill hospitalized children with signs of dehydration over 48 hours were predominantly due to changes in body water rather than tissue deposition. Absolute, percentage and Z score changes in MUAC and WFLz of the children between the first and the second measurements were calculated. Linear regression, adjusted for age and sex was used to quantify changes associated with rehydration.

Four categories of rehydration were defined: less than 0%, 0-4.9%, 5-9.9%, and 10% or more change in body weight. Changes in MUAC and WFLz for children in these categories of dehydration were calculated. Three categories of wasting were defined as severe, moderate and none using MUAC cut offs of < 115 mm, 115 to 124 mm, and 125 mm or more; and using WFLz by <-3, -3 to -2.01, and -2.01 or more. The proportion of children whose anthropometric nutritional classification changed after 48 hours was calculated with 95% confidence intervals (CI).

## Results

### Inter-observer reliability

Sixty children were measured by the two observers. The median and inter-quartile ranges (IQR) were: age 12 months (8 to 20 months), MUAC 130 mm (116 to 148 mm), weight 7.5 kg (6.2 to 9.2 kg) and height/length 70.2 cm (65.1 to 78.4 cm). The inter-observer reliability (ICC) was 0.99 (95% CI 0.97 to 1.0) for MUAC, 0.97 (95% CI 0.85 to 1.0) for weight and 0.99 (95% CI 0.96 to 1.0) for height/length. The ICC values for calculated Z scores were 0.91 (95% CI 0.65 to 1.0) for WFAz, 0.93 (95% CI 0.71 to 1.0) for HFAz and 0.82 (95% CI 0.47 to 1.0) for WFLz.

### Repeated measures study

Between February and August 2009, 382 eligible children were admitted. Forty one had missing information including missing second measures (n = 36) and 5 were missing Z score values for the 1^st ^or 2^nd ^measurement. Eight children died before the 2^nd ^measure and another eight children were hospitalized for less than 2 days. Thus, data on 325 children (61% male) were available for analysis; they had a median age of 11 months (IQR 8 to 18 months).

Of the 325 participants, 261 (80%) had a history of diarrhoea. One hundred and eighty five (57%) had multiple signs of dehydration: 30% had 2 signs, 16% had 3 signs, 5% had 4 signs and 6% experienced 5 or more signs at admission (Table 1). Out of the 325 participants, 37 (11%) had pneumonia, 10 (3.1%) had malaria parasitemia and 9 (2.8%) had a positive HIV antibody test.

**Table 1:** Frequency of signs of dehydration at admission among the 325 study participants

Signs		Frequency	Percentage
**Sunken eyes**		275	86%
**Reduced skin turgor**	**(>2 sec)**	158	49%
**Delayed capillary refill**	**(>2 sec)**	120	37%
	**(> 3 sec)**	29	9.0%
**Deep acidotic breathing**		97	30%
**Palpable limb temperature gradient**		55	17%
**Weak pulse**		33	10%

*Notes*: Children may have had more than one admission diagnosis thus the sum of the cell count exceeds total sample size n = 325

The median MUAC, weight and length among the study participants at admission were 130 mm (IQR 120 mm to 140 mm), 7.3 kg (IQR 6.2 kg to 8.5 kg) and 71.3 cm (IQR 66.7 cm to 76.3 cm) respectively. The median WFAz was -2.1, WFLz was -2.0 and HFAz was -1.3. Seventy eight children (24%) were classified as severely wasted at admission (WFLz <-3).

After 48 hours, the overall mean weight gain was 0.21 kg (95% CI 0.17 to 0.25 kg), corresponding to a mean percentage weight gain of 2.9% (95% CI 2.4 to 3.4%). Whilst the majority n = 243 (75%) of the children gained weight (median 0.26 kg IQR 0.12 to 0.5), 81 children (25%) lost weight (median -0.12 kg, IQR -0.24 to -0.07). Of these 81, 8 (10%) had WFLz <-3 z scores and 9 (11%) had MUAC < 11.5 cm. Ninety one (28%) of the participants gained 5% or more of their admission bodyweight, including 22 (7%) who gained 10% or more. The mean gain in WFLz was 0.29 z scores (95% CI 0.21 to 0.36 z scores), in MUAC was 1.0 mm (95% CI 0.6 to 1.4 mm) and in MUACz was 0.09 z scores (95% CI 0.06 to 0.14 z scores).

In linear regression models, adjusted for age and sex, a one percent (1%) change in weight, was associated with a 0.40 mm (95% CI of 0.30 to 0.44 mm, P < 0.001) change in MUAC, 0.035z (95% CI 0.027 to 0.04z, P < 0.001) change in MUACz and a 0.115z (95% CI of 0.11 to 0.12z, P < 0.001) change in WFLz. In all the models, age had no significant effect; however, there was an effect of sex. There was no evidence of interaction between sex and percent weight gain thus adjusted estimates were not gender stratified in Table 2. Results did not significantly differ when we repeated the analysis after excluding the 81 children who lost weight during the period studied.

**Table 2:** Linear regression model of change in MUAC, MUACz and WFLz with percent change in weight

Predictors of change in MUAC	Coefficient	P value	95% Confidence Interval
**1% change in weight**	**0.359**	0.001	0.28 to 0.44
**Sex (female)**	0.892	0.020	0.14 to 1.64
**Age in months**	-0.019	0.330	-0.06 to 0.02
			
**Predictors of change in MUACz**	**Coefficient**	**P value**	**95% Confidence Interval**

**1% change in weight**	**0.0348**	0.001	0.027 to 0.043
**Sex (female)**	0.079	0.033	0.0064 to 0.152
**Age in months**	-0.002	0.203	-0.006 to 0.001
			
**Predictors of change in WFLz**	**Coefficient**	**P value**	**95% Confidence Interval**

**1% change in weight**	**0.115**	0.001	0.114 to 0.116
**Sex (female)**	-0.024	0.001	-0.344 to -0.013
**Age in months**	- 0.001	0.721	-0.0006 to 0.0004

**Figure 1:**
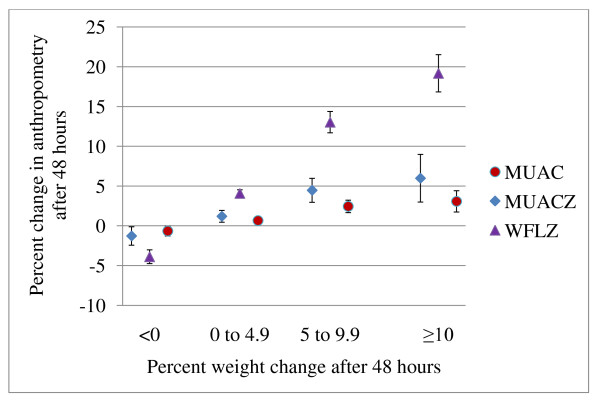
**Percentage change in anthropometry for different categories of percent weight change measured after 48 hours**.

Participants with more than 10% gain in body weight following rehydration, suggesting severe dehydration had been present, had a mean change of +1.4 WFLz and +3.6 mm MUAC. The percent changes on MUAC and WFLz across the different categories of dehydration and their 95% CIs are presented in Figure 1. WFLz had much greater percentage changes, including a 20% change among the most severely dehydrated compared to 6% change by MUACz and 3% change in absolute MUAC.

For analysis of changes in nutritional classification, children were initially included irrespective of whether they gained or lost weight, and if they were 6 to 59 months old (n = 292), since MUAC Cut-off values are currently undefined below this age group. SAM at admission was more commonly identified by WFLz: 71 (24%) children classified as severely malnourished by WFLz at admission compared with 48 (16%) children classified as SAM by MUAC (P < 0.001). Of the 71 children with WFLz<-3, 15 (21%) were not severely malnourished after rehydration. Of the 48 children with MUAC <115 mm, 9 (19%) were not severely malnourished after rehydration. We repeated the analysis excluding the patients who lost weight (n = 218). The proportions misclassified as having severe malnutrition were increased to 32% and 23% for WFLz and MUAC respectively (Table 3).

**Table 3:** Potential misclassification in acute malnutrition among dehydrated children

	ALL PATIENTS (6 to 59 months)			EXCLUDING THOSE WHO LOST WEIGHT		
**MUAC**	**Before**	**After**	**Children that change category**	**Before**	**After**	**Children that change category**

**Severe**	48 (16%)	39 (13%)	-9/48 (-19%)	40(18%)	31(14%)	-9/40 (-23%)
**Mod**	56 (19%)	60 (21%)	+4/56 (+7%)	43(20%)	45 (21%)	+2/43 (+5%)
**Normal**	188 (64%)	193 (66%)	+5/188 (+3%)	135(62%)	142(65%)	+7/135 (+5%)
**Total**	**292 (100%)**	**292(100%)**		**218 (100%)**	**218 (100%)**	
						

**WFLz**	**Before**	**After**	**Children that change category**	**Before**	**After**	**Children that change category**

**Severe**	71 (24%)	56 (19%)	-15/71 (-21%)	62 (28%)	42(19%)	-20/62 (-32%)
**Mod**	80 (27%)	63 (22%)	-17/80 (-21%)	63 (29%)	45 (21%)	-18/63 (-29%)
**Normal**	141 (48%)	173 (59%)	+32/141 (+23%)	93 (43%)	131(60%)	+38/93 (41%)
**Total**	**292 (100%)**	**292(100%)**		**218 (100%)**	**218 (100%)**	

For children classified as moderately malnourished by WFLz <-2, 21% were not moderately wasted after rehydration whereas for children classified as moderately malnourished by MUAC <125 mm, 7% were not moderately malnourished after rehydration (Table 3). After excluding those who lost weight, the proportion of admissions who were classified as moderately malnourished changed to -29% for WFLz and +5% for MUAC.

## Discussion

We have shown that among children admitted to a rural Kenyan district hospital; both MUAC and WFLz were altered by rehydration. Among children who were rehydrated by 10% or more of their admission body weight, the mean change in MUAC was 3.6 mm and in WFLz was 1.4 z scores. We also found that amongst all children with signs of dehydration, approximately one in five children who were classified as severely malnourished at admission by either MUAC or WFLz were classified as non-severely malnourished after 48 hours. Amongst those who gained weight during the 48 hours, approximately one in three became non-severe using WFLz compared to one in four using MUAC.

Since MUAC decreased in children who lost weight and increased proportionately with the degree of rehydration among those who gained weight over a short period of time, we believe that these changes were mostly due to changes in hydration.

There is a paucity of published literature describing changes on diagnostic criteria for SAM among acutely ill hospitalised patients and none has previously reported a repeated measures approach among children. In a study of adults with acute gastroenteritis, anthropometric measures at admission and at 4 weeks post discharge indicated a significant change in both weight and MUAC [18]. Similar to our findings, the authors concluded that the anthropometric changes observed were explained by dehydration and not a change in nutritional status.

Our findings have important implications for the identification and management of individual children with severe malnutrition and for estimations of the prevalence of SAM among hospitalized children. They indicate that such studies may overestimate the true prevalence of SAM and may potentially confound associations if dehydration is also associated with the outcome studied. Since many deaths occur early in admission [19] this is not easily resolved by using later measurements.

A recent study among severely wasted children (6 to 36 months) with cholera in Bangladesh (mean admission WFLz -3.09 and MUAC 11.3 cm) [20] reported an average weight gain of approximately 11% at 72 hours post admission. Anthropometric changes during this period were not evaluated. However, if our findings are generalisable, then the 11% weight gain observed in that study would equate to an increase of ~1.5z scores in WFLz after hydration. Many of the children may not have fulfilled the WHO criteria for severe acute malnutrition at admission had they not been dehydrated.

Our findings may reflect the hospital setting and may differ in a village or community context where both the severity of illness and access to services vary. It is unknown to what extent children with poor fluid intake and ongoing losses might become more dehydrated during a long walk to access clinic services.

In our study, a quarter of the children lost weight suggesting further water loss over the observation period. This did not alter our models for percentage change in anthropometric indices. However, when these children were excluded, the proportion who changed nutritional status classification was markedly increased. The difference was especially evident for moderate malnutrition assessed by WFLz compared to MUAC. It is recognised that signs of dehydration, including sunken eyes and delayed skin pinch which were the most common signs in our study, may be unreliable indicators in malnourished children [21]. It is therefore possible that dehydration could be over-diagnosed among malnourished children. This study was not designed to discover whether this was due to children not taking the prescribed amount of fluid, or whether strategies for rehydration were effective.

A strength of this study was in assessing the inter-observer reliability of the two observers before they were involved in data collection thus increasing the trustworthiness of the results. It is notable that for inter-observer reliability, the lowest ICC estimate was for the WFLz measurements. One limitation of this study is that we used a 2 mm scale MUAC tape instead of a 1 mm graduated tape, which limited the precision of absolute MUAC changes. The use of percentage weight change as a measure of hydration may also be a limiting factor as food, drinks and passing stool or urine shortly before measurement may also have altered weight. More precise estimation of hydration including isotope dilution or bioelectrical impedance methods could be used in future.

## Conclusions

We have shown that overall; MUAC is less affected by dehydration than WFLz. However, changes in classification of severe acute malnutrition following rehydration were similar using the two measures. We recommend assessment of ill children by MUAC because it is affected less by dehydration. Dehydrated children who are classified as severely malnourished should be managed according to protocols specific to malnutrition, including F-75 milk and antimicrobials in addition recommended rehydration fluids and electrolytes, and their anthropometry re-assessed after rehydration. Those who are then found to be only moderately acutely malnourished, and who have regained appetite may then enter a supplementary feeding programme in order to focus inpatient therapeutic resources on children who are most at risk.

## List of abbreviations

MUAC: Mid-Upper Arm Circumference; MUACz: MUAC-for-age Z score; WFLz: Weight-for-Length Z score; WFAz: Weight-for-age Z score; SAM: Severe Acute Malnutrition; WHO: World Health Organisation; UNICEF: United Nations Children's Education Fund; ICC: Intra Class Correlation Coefficient.

## Competing interests

The authors declare that they have no competing interests.

## Authors' contributions

MM designed the study and undertook training and supervision of the study activities, data management, analysis and interpretation of the results and writing the first draft of the manuscript. JB and AP conceived the study, participated in study design and provided overall supervision, advice and expertise on the study. GF provided statistical support and advice on design, analysis and interpretation. All authors were involved in writing, have read and approved the final manuscript.

## Authors' information

MM is a third year PhD student in nutrition epidemiology at the London School of Hygiene and Tropical Medicine, London. This paper forms part of her overall PhD thesis on the usefulness of MUAC among rural community children. GF is the lead statistician at the KEMRI-Wellcome Trust Research Programme, Kilifi, Kenya and has been in post since October 2003. He's interested in wide scale public health interventions. AP and JB jointly supervise MM in all aspects of her work. AP is Head of the MRC International Nutrition Group at LSHTM and MRC Keneba, The Gambia, and specialises in maternal and child nutrition in sub-Saharan Africa. JB is a welcome Trust Intermediate Clinical Research Fellow. His research focuses on interventions and the basic mechanisms involved in the relationship between malnutrition and infection in children so as to improve outcomes in malnourished children.
